# Increased NF-*κ*B DNA binding but not transcriptional activity during apoptosis induced by the COX-2-selective inhibitor NS-398 in colorectal carcinoma cells

**DOI:** 10.1038/sj.bjc.6601266

**Published:** 2003-09-30

**Authors:** H J M Smartt, D J E Elder, D J Hicks, N A Williams, C Paraskeva

**Affiliations:** 1 Cancer Research UK Colorectal Tumour Biology Research Group, Department of Pathology and Microbiology, School of Medical Sciences, University of Bristol, University Walk, Bristol BS8 1TD, UK; 2 Department of Pathology and Microbiology, Division of Immunology, School of Medical Sciences, University of Bristol, University Walk, Bristol BS8 1TD, UK

**Keywords:** NF-*κ*B, COX-2, NSAIDs, apoptosis, colon, therapy

## Abstract

Nonsteroidal anti-inflammatory drugs (NSAIDs) inhibit colorectal neoplasia, an effect that is associated with their ability to induce apoptosis. Although NSAIDs have been reported to inhibit NF-*κ*B, more recent studies show activation of NF-*κ*B by NSAIDs. NF-*κ*B commonly shows antiapoptotic activity and is implicated in the therapeutic resistance of cancer cells. The effects of highly COX-2-selective NSAIDs such as NS-398 on NF-*κ*B in colorectal tumour cells have not been reported. Therefore, we addressed whether NF-*κ*B has a role in NS-398-induced apoptosis of colorectal cancer cells. Treatment of HT-29 colorectal carcinoma cells with doses of NS-398 (50–75 *μ*M) known to induce apoptosis had no effect on NF-*κ*B for up to 48 h. However after 72 and 96 h NF-*κ*B DNA-binding activity was increased by NS-398, in parallel with apoptosis induction. NS-398-treated HT-29 cells showed increased p50 homodimer binding and an induction of p50/p65 heterodimers, as demonstrated by supershift assay. However, although NS-398 increased NF-*κ*B DNA binding it did not increase NF-*κ*B-dependent reporter activity and inhibition of NF-*κ*B DNA binding did not enhance NS-398-induced apoptosis. This indicates that NF-*κ*B activated by NS-398 is transcriptionally inactive and is an encouraging result for the use of COX-2-selective NSAIDs not only in chemoprevention but also as novel therapies for colon cancer.

The term NF-*κ*B refers to a family of dimeric transcription factors binding a common DNA sequence motif known as the *κ*B site. In mammals there are 5 NF-*κ*B family members, p50, p52, p65 (RelA), RelB and c-Rel, which form various homo- and heterodimers. In most resting cell types, NF-*κ*B is sequestered in the cytoplasm via binding to inhibitory proteins of the I*κ*B family. Upon exposure to stimuli such as TNF-*α*, I*κ*B proteins are phosphorylated and degraded, allowing NF-*κ*B proteins to enter the nucleus and bind to *κ*B elements of target gene promoters. NF-*κ*B is considered to have a critical role in the regulation of apoptosis due to its ability to activate the expression of many antiapoptotic genes, for example, TRAF and IAP proteins, c-FLIP, Bcl-X_L_ and A1 (reviewed in Karin *et al*, 2002; Karin and Lin, 2002).

NF-*κ*B activity varies within the colonic crypt and has been implicated in the maintenance of colonic crypt homeostasis (Inan *et al*, 2000), which relies on tight regulation of proliferation, differentiation and apoptosis (Williams *et al*, 1996). Moreover, NF-*κ*B activity may be deregulated in colorectal cancer (Lind *et al*, 2001). NF-*κ*B has been implicated in the resistance of colon cancer cells to therapeutic agents. For example, the active metabolite (SN38) of the colon cancer therapeutic irinotecan (CPT-11) was shown to activate NF-*κ*B in most colorectal cell lines *in vitro.* Blocking activation of NF-*κ*B enhanced both the cytotoxicity of SN38 *in vitro* and the sensitivity of tumours to CPT-11 in a murine colorectal cancer xenograft model (Cusack *et al*, 2000).

Nonsteroidal anti-inflammatory drugs (NSAIDs) show antineoplastic activity in the colon which appears to be due, at least in part, to NSAID-induced apoptosis (Shiff *et al*, 1996; Elder *et al*, 1997; Piazza *et al*, 1997). Large population-based studies suggest an approximately 40–50% reduction in relative risk of developing colon cancer by chronic use of NSAIDs such as aspirin. Moreover, sulindac reduces the size and number of colonic polyps in familial adenomatous polyposis (FAP) patients (reviewed in Gupta and DuBois, 2001). However, chronic use of these traditional NSAIDs can cause gastrointestinal side effects such as bleeding and ulceration, limiting their widespread therapeutic use against colon cancer. Gastrointestinal side effects of NSAIDs have been linked to a lack of selectivity in their inhibition of cyclo-oxygenase (COX) isoforms: traditional NSAIDs inhibit the constitutive, ‘housekeeping’ COX-1 isoform in addition to the inducible, inflammation-associated COX-2 isoform. This led to the development of gastrointestinal-sparing COX-2-selective NSAIDs, such as celecoxib. Like sulindac, celecoxib also causes a reduction in the number of colorectal polyps in FAP patients (Steinbach *et al*, 2000) and has been approved in the USA as an adjunct to standard care in this disease. Although NSAIDs are currently being evaluated for their effectiveness in the chemoprevention of colon cancer, there is also increasing interest in their possible use in colon cancer therapy, either in combination with existing therapeutic agents or as novel therapeutic agents (Tebbutt *et al*, 2002; Trifan *et al*, 2002).

Although the best-characterised target of NSAIDs is COX, several lines of evidence suggest additional, COX-independent targets such as NF-*κ*B (Shiff and Rigas, 1999). Although several studies report the inhibition of stimulus-induced NF-*κ*B by some NSAIDs, namely aspirin, salicylate and sulindac, this is not a property of all NSAIDs (Kopp and Ghosh, 1994; Yamamoto *et al*, 1999). Interestingly, several more recent papers report activation of NF-*κ*B by NSAIDs (Niederberger *et al*, 2001; Stark *et al*, 2001; Moalic-Juge *et al*, 2002). Hence, it appears that NSAIDs can either inhibit or activate NF-*κ*B. NF-*κ*B has an important role in the regulation of apoptosis, usually showing antiapoptotic activity. Therefore, inhibition of NF-*κ*B by NSAIDs could theoretically contribute to their induction of apoptosis; conversely activation of NF-*κ*B could potentially limit the apoptotic response. To our knowledge no one has addressed the effect of a COX-2-selective NSAID on NF-*κ*B during NSAID-induced apoptosis of colorectal cancer cells. Therefore, we examined whether NF-*κ*B activity was either inhibited or increased by NS-398 treatment and, if so, whether it had a role in NS-398-induced apoptosis of colorectal cancer cells. Since we are specifically interested in the potential role of NF-*κ*B in NS-398-induced apoptosis, as well as examining cells at early time points (1–24 h) normally associated with NF-*κ*B activation, later time points were also examined (48–96 h). This is because apoptosis induced by a number of reagents occurs maximally between 48 and 96 h in colorectal cancer cells (Bracey *et al*, 1995; Elder *et al*, 1996). These studies could have important implications for the use of COX-2-selective inhibitors not only in chemoprevention but also as novel therapies for colorectal cancer, where they may be used as single agents or in combination.

## MATERIALS AND METHODS

### Cell culture and treatment

HT-29 colon carcinoma cells, which express detectable levels of COX-2 protein (Elder *et al*, 1997), were grown as an adherent monolayer in T25 tissue culture flasks in DMEM supplemented with 10% foetal bovine serum, penicillin (100 U ml^−1^), streptomycin (100 *μ*g ml^−1^) and glutamine (2 mM). The NSAID NS-398 is highly selective for inhibition of COX-2 (Warner *et al*, 1999) and has previously been used to investigate the effects of COX-2-inhibition *in vitro* (Tsujii *et al*, 1998). NS-398 (Cayman, Ann Arbor, MI, USA) was made up as a 30 mM stock solution in DMSO and stored at −20°C. Tumour necrosis factor (TNF)-*α* (Autogen Bioclear, Wiltshire, UK) was made up as a 100 *μ*g ml^−1^ aqueous stock solution and stored at –20°C. For treatment with NS-398 or TNF-*α*, cells were harvested by trypsinisation (0.1% w v^−1^ trypsin/EDTA), seeded at 0.5 × 10^6^ cells per flask and cultured for 4 days prior to treatment. Unless otherwise stated, cell cultures were treated in triplicate with NS-398 for up to 96 h or with TNF-*α* for 24 h (as TNF-*α* is a more rapid inducer of apoptosis than NS-398). Each control and treated flask received an equal final volume of vehicle, to a maximum of 0.01% (v v^−1^) for tissue culture water and 0.25% (v v^−1^) for DMSO (at this concentration DMSO does not affect cell viability, data not shown). NS-398 was used between 50 and 75 *μ*M, as these doses induce apoptosis in HT-29 cells (Elder *et al*, 1997); lower doses generally do not induce apoptosis in colorectal cell lines (Elder *et al*, 2000) and higher doses (e.g. 100 *μ*M) induced a degree of nonapoptotic toxicity in HT-29 cells after 72 h (data not shown) and were therefore not routinely used. TNF-*α*, a well characterised inducer of anti-apoptotic NF-*κ*B activity (reviewed in Karin and Lin, 2002), was used as a control for apoptosis experiments at 100 ng ml^−1^, a dose used by other investigators in this cell type (Giardina *et al*, 1999). For reporter assays, the dose of TNF-*α* was reduced to 1 ng ml^−1^, in order to activate a similar level of DNA binding activity to that seen with NS-398 treatment. Cells were harvested at the indicated times for the preparation of nuclear protein extracts or passive lysis buffer (PLB) lysates (see below), or for cell counts. For the latter, the attached cells (those adhered to the tissue culture flask) and the floating cells (those having detached from the adhered monolayer) were harvested and counted separately using a haemocytometer. Separate aliquots of attached and floating cells were stained with acridine orange for analysis by fluorescence microscopy (see below).

### Measurement of apoptosis

We and others have previously shown that in routine culture of colorectal epithelial tumour cells, the majority of spontaneously occurring floating cells are apoptotic, while the proportion of attached cells that are apoptotic is low (<3%) (Hague *et al*, 1993). Treatment with agents such as NSAIDs does not generally increase the proportion of attached cells with apoptotic morphology but does increase the proportion of floating cells, and this is due to induction of apoptosis (Elder *et al*, 1996, 1997). Hence, in these cases, the level of apoptosis in cultured epithelial cells can be determined by measuring the proportion of the total cell population that has detached from the cell monolayer. In this study, apoptosis induced by NS-398 was determined as described previously by Hague *et al* (1993). Briefly, after determining the proportion of cells that had detached from the cell monolayer and were floating in the medium, the fraction of these ‘floating’ cells that were apoptotic was assessed by morphology following acridine orange staining. Biochemical confirmation of apoptosis was obtained by demonstration of PARP cleavage by Western blotting. For this purpose attached cells were harvested at 96 h; floating cells were harvested at 48 h and again at 96 h to minimise further degradation of the cleaved PARP protein.

### Preparation of nuclear protein extracts

Nuclear protein extracts were prepared using a protocol based on the method of Andrews and Faller (1991). Cell cultures were washed twice and scraped into ice-cold phosphate-buffered saline (PBS). All subsequent stages were carried out at 4°C or on ice. Cells were centrifuged (10 000 **g**, 2 min) and pellets resuspended in ice-cold buffer 1 (10 mM HEPES–KOH pH 7.9, 10 mM KCl, 1.5 mM MgCl_2_, 1 mM DTT, 0.5 mM PMSF and 2 *μ*g ml^−1^ leupeptin). Cells were incubated for 10 min to allow swelling and then lysed by vigorous vortexing for 30 s. After addition of NP-40 (final concentration 3.75%) and a further minute of vortexing, nuclei were pelleted by centrifugation (10 000 **g**, 1 min). Nuclei were resuspended in ice-cold buffer 2 (20 mM HEPES–KOH pH 7.9, 400 mM NaCl, 1.5 mM MgCl_2_, 0.2 mM EDTA, 25% glycerol, 1 mM DTT, 0.5 mM PMSF and 2 *μ*g ml^−1^ leupeptin) and incubated on ice for 20 min. After centrifugation (10 000 **g**, 2 min), the supernatant (nuclear protein extract) was removed and aliquots stored at −70°C. Protein concentrations of the nuclear protein extracts were determined using the Bradford-based BioRad protein assay (BioRad Laboratories, Hercules, CA, USA).

### Electrophoretic mobility shift assay (EMSA)

To assay DNA-binding by EMSA a double-stranded NF-*κ*B-binding oligonucleotide (AGTTGAGGGGACTTTCCCAGGC; Promega, Southampton, UK) was end-labelled with [*γ*−^32^P]ATP using T4 polynucleotide kinase (Boehringer Mannheim, Germany). Binding reactions were performed in a total volume of 20 *μ*l Roche, East Sussex, UK containing 1 mM MgCl_2_, 1 mM KCl, 7.5% (w v^−1^) Ficoll, 8.5 mM HEPES, 1 mM DTT, 1 mg ml^−1^ BSA, 1–4 *μ*g poly(dI-dC) (Roche East Sussex, UK) and 1–4 *μ*g nuclear protein extract. Reactions were incubated for 15 min at room temperature, during which time an excess of unlabelled NF-*κ*B oligonucleotide (see above) or unlabelled AP-1 oligonucleotide (CGCTTGATGAGTCAGCCGGAA, Promega, UK) was added where appropriate for competition assays, followed by a 5 min incubation. A volume of 2 *μ*l of end-labelled NF-*κ*B oligonucleotide at 20 000 cpm *μ*l^−1^ was then added and, following a further 20 min room temperature incubation, protein–DNA complexes were separated from free oligonucleotide on a 5% nondenaturing polyacrylamide/Tris borate EDTA gel at 150 V for 2 h and visualised by autoradiography. For supershift assays, 1 *μ*l of NF-*κ*B antibody or control IgG was preincubated for 30 min at room temperature with the nuclear extract-binding mix prior to addition of labelled NF-*κ*B oligonucleotide. Rabbit anti-NF-*κ*B antibodies for supershift analysis (p50: sc-114; p65: sc-372; p52: sc-298; c-Rel: sc-70 and RelB: sc-226) and control IgG were all obtained from Santa Cruz Biotechnology (USA).

### Protein extracts and SDS–PAGE Western blotting

For Western blotting, cells were harvested by trypsinisation (0.1% w v^−1^ trypsin/EDTA) and counted using a haemocytometer. Western samples were prepared from 10^6^ adherent cells (for I*κ*B*α*) or from 2 × 10^6^ each of adherent and floating cells (for PARP cleavage). Cellular protein lysates were prepared in sample buffer (62 mM Tris-HCl (pH 6.8), 10% (v v^−1^) glycerol, 5% (v v^−1^) 2-mercaptoethanol, 4% (w v^−1^) SDS, 0.01% (w v^−1^) bromophenol blue) and SDS–PAGE immunoblotting carried out as described previously (Elder *et al*, 2000). I*κ*B*α* protein was detected using polyclonal anti-I*κ*B*α* at 1 : 1000 (NEB, Beverly, MA, USA). Full-length PARP protein (118 kDa) and the 85 kDa apoptosis-induced cleavage product were detected using monoclonal anti-PARP at 1 : 1000 (Alexis Biochemicals, Nottingham, UK). Loading and transfer were controlled by repeat probing with anti-*α*-tubulin (Sigma, Dorset, UK) at 1 : 20 000. Membranes were subsequently probed with an anti-rabbit (for I*κ*B*α*) or an anti-mouse (for PARP and *α*-tubulin) secondary antibody conjugated to horseradish peroxidase (Sigma) at 1 : 1000 or at 1 : 2000 for use following anti-*α*-tubulin. Bound antibodies were detected using enhanced chemiluminescence (Kirkegaard and Perry, Gaithersburg, MD, USA). If necessary, membranes were stripped by incubation for 30 min at 50°C in a solution of 62 mM Tris-HCl pH 6.7, 2% SDS and 100 mM 2-mercaptoethanol.

### Adenovirus-driven overexpression of I*κ*B*α*

A recombinant adenovirus (rAd.I*κ*B*α*) carrying the wild-type porcine I*κ*B*α* gene fused to a nuclear localisation signal was used to overexpress I*κ*B*α* protein in HT-29 cells (Wrighton *et al*, 1996). Porcine I*κ*B*α* is strongly homologous to human I*κ*B*α* (de Martin *et al*, 1993) and this adenoviral vector has previously been shown to inhibit activation of NF-*κ*B in human cells (Liu *et al*, 2001). rAd.I*κ*B*α* was a kind gift from Dr Ranier de Martin (Department of Vascular Biology and Thrombosis Research, University of Vienna, Austria). As a control for infection experiments, a recombinant adenovirus carrying the bacterial LacZ gene (rAd.*β*gal) was used (Wilkinson and Akrigg, 1992). rAd.*β*gal was a kind gift from Dr G Wilkinson (Department of Cardiology and Medicine, University of Wales College of Medicine, Heath Park, Cardiff, UK). Cells were infected with viral vectors in normal growth medium for 4 h and then allowed to recover overnight in normal growth medium prior to experimental treatment the following day.

### Cell transfection

For NF-*κ*B reporter assays, cells were transiently transfected with either the NF-*κ*B reporter plasmid pNF-*κ*B-TA-luc or with the control reporter plasmid pTA-luc (Clontech, Oxford, UK). pNF-*κ*B-TA-luc contains four copies of a consensus NF-*κ*B binding sequence (GGGAATTTCC) in addition to a minimal promoter (P_TA_, the TATA box from the herpes simplex virus thymidine kinase promoter) located upstream of the firefly luciferase (*luc*) gene. The consensus NF-*κ*B binding sequences are absent from the control vector pTA-luc. All transfections also included the renilla luciferase vector pRL-SV40 (Promega, UK) as an internal control for transfection efficiency.

For transient transfection, HT-29 cells were seeded at 0.5 × 10^6^ cells per T25 flask. At 3 days after seeding, triplicate flasks were cotransfected with one of the two reporter constructs (pTA-luc or pNF-*κ*B-TA-luc) and with the renilla construct (pRL-SV40) in a ratio of 50 : 1. Each flask was incubated for 6 h with 2 *μ*g of plasmid DNA and 5 *μ*l of lipofectAMINE 2000 (Invitrogen) diluted in opti-MEM serum-free medium according to manufacturers' instructions. Following transfection, cells were allowed to recover overnight in 10% DMEM prior to experimental treatment as described above. A dose of 75 *μ*M NS-398 was used for reporter assays in order to maximise the activation of NF-*κ*B DNA binding in these experiments.

### Luciferase reporter assay

At the specified time, cells were washed in PBS and lysates prepared in 1 × PLB Promega, UK) according to manufacturers' instructions. Reporter activity was measured using the Dual-Luciferase reporter assay system (Promega, UK) and a Jade Luminometer (Labtech, UK) set for a 10 s read. Sample readings were corrected for background autoluminescence using untransfected cells as a control.

## RESULTS

### NS-398 increases NF-*κ*B DNA-binding at late but not early time points in colorectal cancer cells

**Figure 1:**
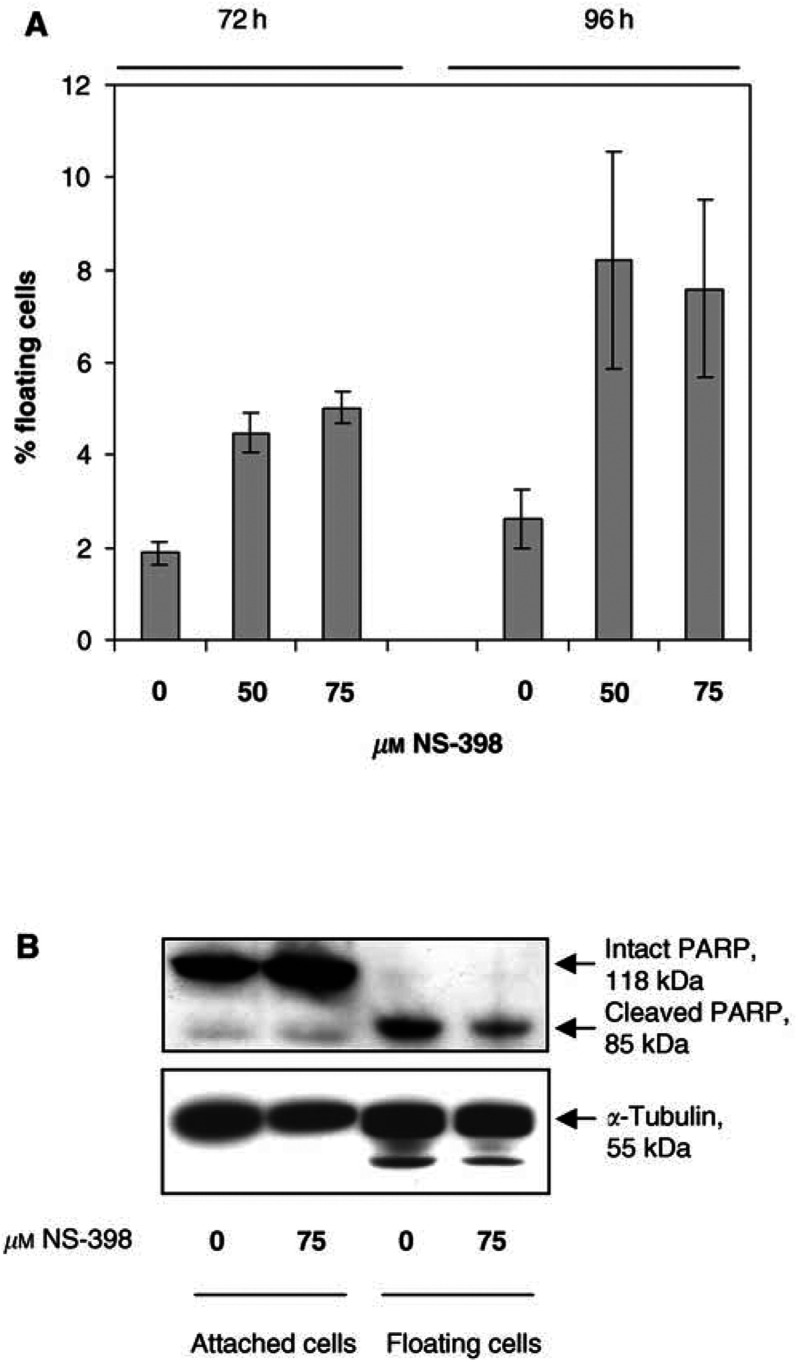
Induction of apoptosis by the COX-2-selective NSAID NS-398 in colorectal carcinoma cells. (**A**) *N-398 induces apoptosis in HT-29 cells.* HT-29 cells were treated with 0 *μ*M (vehicle only), 50 *μ*M or 75 *μ*M NS-398. At 72 and 96 h, both attached and floating cells were harvested and counted and floating cell yields were calculated as a percentage of total cell yields. Floating cell yield data are means±s.e.m. of three independent repeat experiments carried out in triplicate. Apoptotic morphology of floating cells was confirmed by acridine orange staining (as in Elder *et al*, 1997). (**B**) Floating cells show the absence of intact PARP and the presence of cleaved PARP, a biochemical marker of apoptosis. HT-29 cells were treated with 0 *μ*M (vehicle) or 75 *μ*M NS-398 for 96 h. Lysates from 2 × 10^6^ attached and floating cells were analysed by Western blotting for PARP expression and cleavage.

Since NF-*κ*B can limit the response to therapeutic apoptosis-inducing agents, we determined whether NF-*κ*B was activated or inhibited during NS-398-induced apoptosis in colorectal cancer cells. Preliminary experiments were performed to confirm that NS-398 induces apoptosis in HT-29 cells, as previously reported (Elder *et al*, 1997). Doses of 50 and 75 *μ*M NS-398 increased the proportion of HT-29 cells that were detached and floating in the culture medium at 72 and 96 h (Figure 1A

), with no significant effect at earlier time points (data not shown). Our previous studies have shown that these floating cells are apoptotic (Hague *et al*, 1993; Elder *et al*, 1997). The apoptotic nature of floating cells from control and NS-398-treated cultures was confirmed by acridine orange staining as in Elder *et al* (1997) (data not shown). Further confirmation of apoptosis was obtained by Western blotting for PARP protein, a target of activated caspases during apoptosis. Attached (nonapoptotic) cells contained intact 118 kDa PARP protein. However, floating cells showed no evidence of intact PARP, containing only the cleaved 85 kDa PARP fragment, a characteristic marker of apoptosis (Figure 1B).

**Figure 2:**
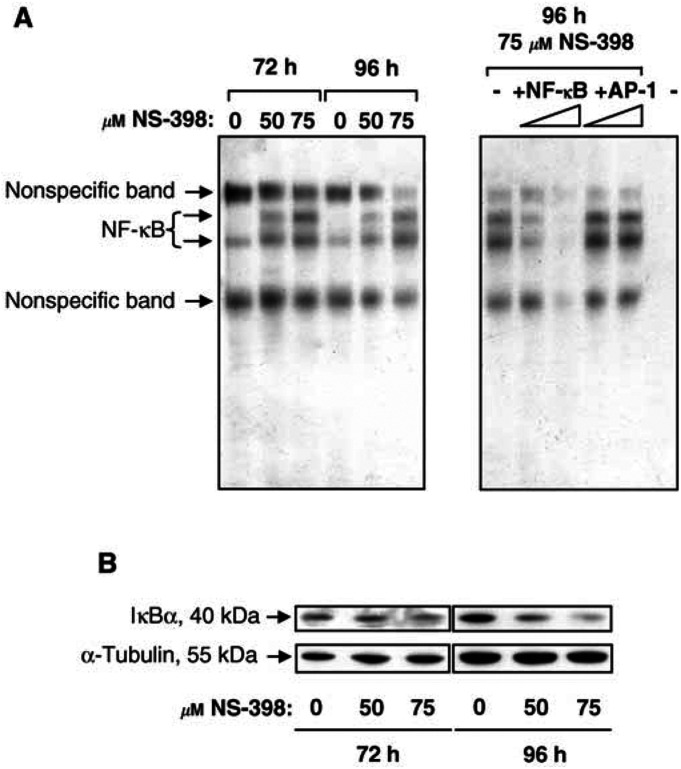
Activation of NF-*κ*B DNA-binding activity in colorectal carcinoma cells by the COX-2-selective NSAID NS-398. (**A**) NS-398 induces NF-κB DNA-binding activity in HT-29 cells. HT-29 cells were treated with 0, 50 or 75 *μ*M NS-398 for 72 or 96 h. At the indicated time points, nuclear extracts were prepared and 1 *μ*g analysed by EMSA. Competition assay (right-hand panel): +NF-*κ*B=plus unlabelled NF-*κ*B oligonucleotide (specific competitor), +AP-1=plus unlabelled AP-1 oligonucleotide (nonspecific competitor), −=no oligonucleotide. The final lane contains no nuclear protein extract as a negative control. (**B**) NS-398 downregulates I*κ*B*α* levels in HT-29 cells. HT-29 cells were treated with 0, 50 or 75 *μ*M NS-398 for 72 or 96 h. Whole-cell lysates from 10^6^ attached cells were analysed by Western blotting for I*κ*B*α* expression levels at 72 and 96 h. Blots were probed with an *α*-tubulin antibody as a control for equal loading and transfer. Results shown are representative of those obtained in three independent repeat experiments.

**Figure 3:**
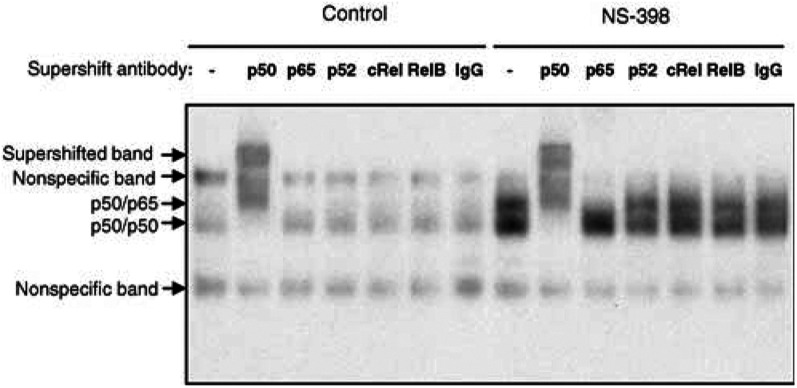
The COX-2-selective NSAID NS-398 induces NF-*κ*B DNA-binding activity in the form of p50/p50 and p50/p65 in HT-29 cells. HT-29 cells were treated with 0 or 50 *μ*M NS-398 for 96 h. Nuclear extracts were prepared and 4 *μ*g analysed by supershift EMSA in the presence or absence of antibodies to NF-*κ*B proteins p50, p65, p52, c-Rel or RelB or control IgG. The p50 antibody supershifts both bands, whereas the anti-p65 antibody only affects the upper band, suggesting that the lower band consists of p50/p50 and the upper band of p50/p65. The p65 antibody does not produce a supershifted band, presumably due to interference with DNA binding. This experiment has also been carried out using samples treated with 60 *μ*M NS-398 for 72 h with equivalent results.

Having confirmed that NS-398 induces apoptosis of HT-29 cells in this system, nuclear extracts were prepared from these cultures and the levels of NF-*κ*B DNA-binding activity were investigated. No effect of up to 100 *μ*M NS-398 on NF-*κ*B DNA-binding was observed at 1, 6, 12 or 24 h and NS-398 did not alter NF-*κ*B-dependent reporter activity after 24 h of treatment (data not shown). However, NF-*κ*B DNA binding was increased by 50 and 75 *μ*M NS-398 at 72 and 96 h (Figure 2A

, left panel), when NS-398-induced apoptosis is maximal. NF-*κ*B DNA-binding was inhibited by an excess of unlabelled NF-*κ*B oligonucleotide but not by unlabelled AP-1 oligonucleotide (Figure 2A, right panel). Concentration not a volume 50 and 75 *μ*M NS-398 did not increase NF-*κ*B DNA-binding at 48 h (data not shown), suggesting that NF-*κ*B is not a cause of NS-398-induced apoptosis. Activation of NF-*κ*B at 72 and 96 h was associated with a slight but reproducible decrease in I*κ*B*α* levels at 72 h and a marked decrease after 96 h of NS-398 treatment (Figure 2B). In order to determine the subunit composition of NF-*κ*B activated by NS-398, supershift EMSA analysis was carried out. NF-*κ*B DNA-binding activity in controls was diminished in intensity and supershifted only by anti-p50 antibodies (Figure 3

), indicating a composition of p50/p50 dimers. NS-398 enhanced p50/p50 NF-*κ*B DNA-binding activity and induced a second, more slowly migrating band. The latter was decreased in intensity on incubation with both anti-p50 and anti-p65 antibodies (Figure 3), indicating that this upper band represented p50/p65 heterodimers (this particular anti-p65 antibody did not produce a supershifted band, presumably due to interference with binding of p65-containing NF-*κ*B to labelled oligonucleotide). Similar results were obtained in supershift analysis of 72 h samples treated with 60 *μ*M NS-398, where a lower p50/p50 band and an upper p50/p65 band were detected. Again control samples contained only p50/p50 activity (data not shown). Further evidence for the involvement of p50 and p65 subunits was obtained using an ELISA-based method (see, for example, Bochkov *et al*, 2002). This showed increased binding of p50 and p65 to an NF-*κ*B consensus oligonucleotide in NS-398-treated HT-29 cells (data not shown).

In summary, the COX-2-selective NSAID NS-398 has no effect on NF-*κ*B DNA-binding activity between 1 and 48 h, suggesting that NF-*κ*B is unlikely to be a cause of NS-398-induced apoptosis (observed maximally between 72 and 96 h). However, NF-*κ*B DNA-binding (p50/p50 and p50/p65) was increased in NS-398 treated cells at 72 and 96 h: this could potentially limit the induction of apoptosis by NS-398.

### Inhibition of NF-*κ*B activation following NS-398 treatment does not enhance apoptosis in HT-29 cells

**Figure 4:**
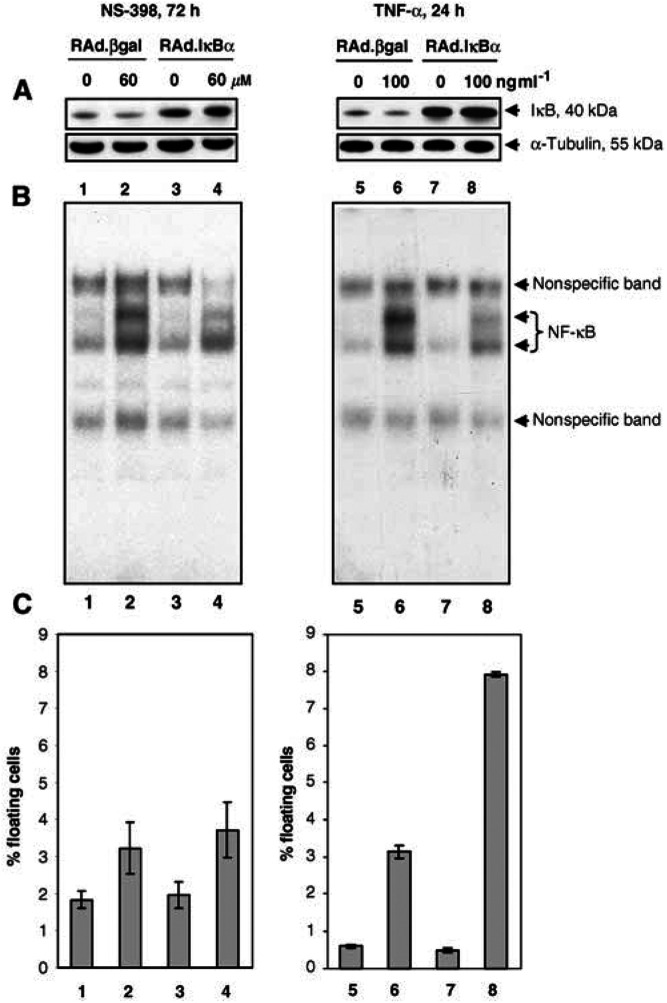
Partial inhibition of NS-398-induced NF-*κ*B DNA-binding activity does not enhance apoptosis induction in HT-29 cells. HT-29 cells were infected with 10 MOI (for NS-398 experiments; left-hand panel) or 50 MOI (for TNF-*α* experiments; right-hand panel) of either the control vector rAd.*β*gal or rAd.I*κ*B*α* and treated the following day with 60 *μ*M NS-398 for 72 h or 100 ng ml^−1^ TNF-*α* for 24 h (as TNF-*α* is a more rapid inducer of apoptosis). (**A**): Increased expression of IκBα in HT-29 cells infected with rAdI*κ*B*α*. Whole cell lysates from 10^6^ attached cells were prepared and analysed by Western blotting for I*κ*B*α* expression levels. Blots were probed with an *α*-tubulin antibody as a control for equal loading and transfer. (**B**): Inhibition of NF-*κ*B DNA binding by rAdI*κ*B*α*. Nuclear protein extracts were prepared and 1 *μ*g analysed by EMSA. (**C**): Infection with rAd.I*κ*B*α* potentiates apoptosis induced by TNF-*α* but not NS-398. Both attached and floating cells were harvested and counted. Floating cell yields were calculated as a percentage of total cell yields and are shown as mean±s.d. Results shown in panels A–C for NS-398 and for TNF-*α* are from within the same experiment and are representative of two independent experiments carried out in duplicate. 1=rAd.*β*gal; 2=rAd.*β*gal+NS-398; 3=rAd.I*κ*B*α*; 4=rAd.I*κ*B*α* +NS-398; 5=rAd.*β*gal; 6=rAd.*β*gal+TNF-*α*; 7=rAd.I*κ*B*α*; 8=rAd.I*κ*B*α* +TNF-*α*.

Activation of NF-*κ*B is reported to limit the efficacy of apoptosis induction by a number of anticancer drugs (reviewed in Barkett and Gilmore, 1999). We next investigated whether blocking the activation of NF-*κ*B (using the adenoviral I*κ*B*α* vector rAd.I*κ*B*α*) sensitised colorectal cells to induction of apoptosis by NS-398. For NS-398 experiments, cells were infected with rAd.I*κ*B*α* at the maximum titre that did not itself result in activation of NF-*κ*B at 72 h, which was 10 MOI. A dose of 60 *μ*M NS-398 was used, as preliminary experiments suggested that this gave optimal apoptosis induction in virus-infected cells. Infection of HT-29 cells with rAd.I*κ*B*α* increased I*κ*B*α* expression (Figure 4A

, lanes 3 and 4) and gave a partial inhibition of NF-*κ*B activity induced by NS-398 (Figure 4B, compare lane 4 with lane 2). However, infection with rAd.I*κ*B*α* did not increase the levels of apoptosis observed in NS-398-treated HT-29 cells compared to infection with the control vector rAd.*β*gal (Figure 4C, compare bar 4 with bar 2).

Inhibition of NF-*κ*B has been shown to enhance TNF-*α*-induced cytotoxicity in a number of cell types, including intestinal epithelial cells (Han *et al*, 2000; Potoka *et al*, 2000). Therefore, in order to determine whether a partial inhibition of NF-*κ*B as observed in NS-398 experiments was sufficient to enhance apoptosis, control experiments were carried out using a second inducer of NF-*κ*B, TNF-*α*. A dose of 50 MOI rAd.I*κ*B*α* was used in order to give a similar degree of inhibition of NF-*κ*B and increase in I*κ*B*α* expression to those seen in NS-398 experiments. Infection of HT-29 cells with rAd.I*κ*B*α* increased I*κ*B*α* expression (Figure 4A, lanes 7 and 8) and gave a partial inhibition of NF-*κ*B activity induced by TNF-*α* (Figure 4B, compare lane 8 with lane 6). Infection with rAd.I*κ*B*α* did not increase the levels of apoptosis observed in untreated HT-29 cells compared to infection with the control vector rAd.*β*gal (Figure 4C, compare bar 7 with bar 5). However, infection with rAd.I*κ*B*α* enhanced apoptosis induced by TNF-*α* approximately two-fold (range 1.9- to 2.5-fold) (Figure 4C, compare bar 8 with bar 6).

### NS-398-induced NF-*κ*B is not active at the level of transcription

**Figure 5:**
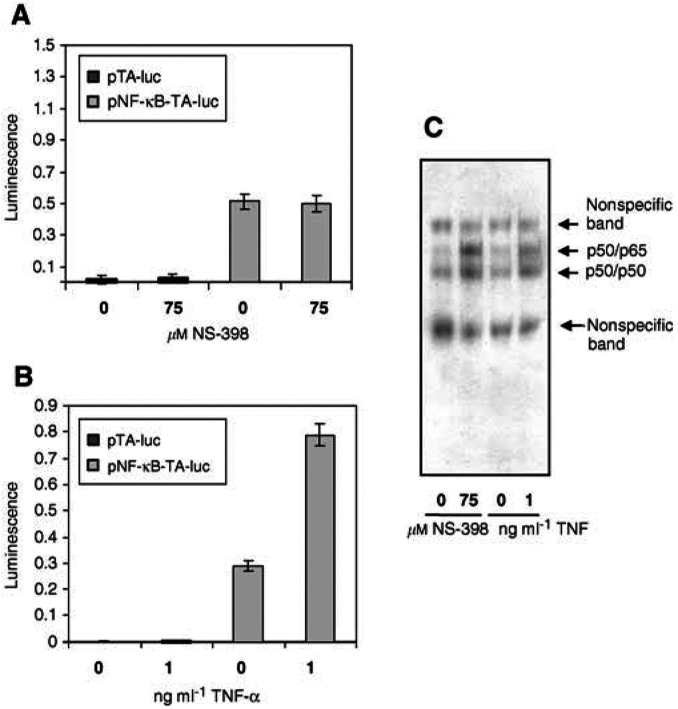
NS-398-induces NF-*κ*B DNA-binding activity but not transcriptional activity in HT-29 cells. HT-29 cells were transfected with either the firefly luciferase reporter vector pNF-*κ*B-TA-luc or with the control vector pTA-luc lacking NF-*κ*B binding sites. All transfections also included the constitutively expressed renilla luciferase vector pRL-SV40 and hence firefly luciferase readings were normalised to renilla luciferase activity in order to control for transfection efficiency. (**A**) NS-398 does not increase NF-*κ*B-dependent transcriptional activity in HT-29 cells. Cells transfected with pTA-luc or pNF-*κ*B-TA-luc were then treated with 0 (vehicle) or 75 *μ*M NS-398 for 72 h. After this time, PLB lysates, were prepared and assayed for firefly and renilla luciferase activities. Luminescence data are expressed as mean firefly:renilla luciferase ratios±s.d. of three independent experiments and within each experiment samples were assayed in triplicate. (**B**) and (**C**) TNF-*α* activates NF-*κ*B-dependent transcriptional activity at doses inducing a similar NF-*κ*B DNA-binding activity to 75 *μ*M NS-398 in HT-29 cells. (**B**) Cells transfected with pTA-luc or pNF-*κ*B-TA-luc were treated with a range of doses of TNF-*α* for 24 h. After this time lysates were prepared and assayed for firefly and renilla luciferase activities. Luminescence data are expressed as mean firefly : renilla luciferase ratios±s.d. from one representative of three independent experiments carried out in triplicate (means were not taken in this case due to different activities between batches of TNF-*α* necessitating the use of differing doses of TNF-*α* between experiments). (**C**) Nuclear protein extracts (from pNF-*κ*B-TA-luc-transfected cells) were prepared using parallel flasks to those from (**A**) and (**B**) and analysed by EMSA for each of the three independent experiments.

One possible reason why inhibition of NF-*κ*B did not enhance NS-398-induced apoptosis is that NF-*κ*B activated by NS-398 is transcriptionally inert. In order to test this hypothesis, NF-*κ*B reporter assays were carried out. HT-29 cells were transiently transfected with either the NF-*κ*B-luciferase reporter pNF-*κ*B-TA-luc or with a control vector lacking NF-*κ*B sites, pTA-luc. Reporter activity in HT-29 cells transfected with pTA-luc was consistently of a very low level (Figure 5A and B

, first two bars). HT-29 cells transfected with pNF-*κ*B-TA-luc showed some constitutive NF-*κ*B reporter activity (Figure 5A and B, third bar from left), consistent with the NF-*κ*B DNA-binding activity observed under these conditions (Figure 5C, first and third lanes). However, treatment of HT-29 cells with NS-398 did not increase NF-*κ*B-dependent reporter activity (Figure 5A, final bar). This was in contrast to TNF-*α* which, at doses inducing a similar increase in DNA-binding activity (Figure 5C, final lane), gave more than a two-fold increase (range 2.5- to three-fold) in NF-*κ*B-dependent transcription (Figure 5B, final bar).

In conclusion, NS-398, unlike TNF-*α*, does not increase NF-*κ*B DNA binding at early time points (1–48 h) but does at the later time points of 72 and 96 h. However, although NS-398 increases NF-*κ*B DNA binding at the later time points, it does not increase NF-*κ*B-dependent reporter activity and inhibition of NF-*κ*B DNA-binding does not enhance NS-398-induced apoptosis, indicating that NF-*κ*B activated by NS-398 is transcriptionally inert and is not involved in NS-398-induced apoptosis.

## DISCUSSION

Although NSAIDs have recently received much attention as promising colon cancer chemopreventives, increasing evidence indicates that they may also be used as a novel therapy, either alone or in combination with other agents (Tebbutt *et al*, 2002; Trifan *et al*, 2002). This stems from the ability of NSAIDs to inhibit invasion and angiogenesis and induce apoptosis, indicating the importance of COX-2 in metastasis (reviewed in Cao and Prescott, 2002). Their use in a therapeutic setting would justify the use of relatively high doses of NSAIDs, which are believed to have both COX-dependent and COX-independent effects (reviewed in Turini and DuBois, 2002). It is of interest to note that a significant number of colorectal tumours do not express COX-2 protein and therefore COX-2-independent effects of NSAIDs could be particularly important in this group of tumours (Elder and Paraskeva, 1998).

The induction of apoptosis is an important mechanism of action of anticancer agents and activation of NF-*κ*B by such agents can contribute to therapeutic resistance of cancer cells. Nonsteroidal anti-inflammatory drugs induce apoptosis in colon cancer cell lines both *in vivo* and *in vitro*. Therefore, *in vitro* induction of apoptosis can be used to highlight key pathways involved in the apoptotic response of colorectal cancer cells to COX-2-selective NSAIDs (Elder *et al*, 2002). Although NSAIDs have been reported to inhibit NF-*κ*B (Kopp and Ghosh, 1994; Yamamoto *et al*, 1999), more recent studies report activation of NF-*κ*B by NSAIDs (Niederberger *et al*, 2001; Stark *et al*, 2001; Moalic-Juge *et al*, 2002). To our knowledge there are no data concerning the effect of highly COX-2-selective NSAIDs on NF-*κ*B activity in colorectal cancer cell lines. Due to our specific interest in the potential role of NF-*κ*B in NS-398-induced apoptosis, which is maximal between 72 and 96 h, we examined these later time points in addition to earlier time points (1–24 h) normally associated with NF-*κ*B activation. The results presented in this study indicate that NS-398 treatment causes a delayed activation of NF-*κ*B DNA-binding, but not NF-*κ*B-dependent transcriptional activity in colon cancer cells. This is based on the following observations: treatment of colorectal cells with NS-398 causes a delayed and sustained activation of NF-*κ*B DNA-binding activity in parallel with the induction of apoptosis. However, the inhibition of NF-*κ*B DNA binding did not enhance NS-398-induced apoptosis of colon cancer cells. Consistent with this finding, NS-398 treatment did not drive transcription from an NF-*κ*B-dependent reporter construct. In contrast to NS-398, the well-characterised cytokine TNF-*α* induced both NF-*κ*B DNA binding and transcriptional activity, which protected TNF-*α*-treated cells against apoptosis induction.

The mechanism by which NS-398 activates NF-*κ*B DNA binding in HT-29 cells is not clear. However, NS-398 is known to activate the MEK/ERK pathway in HT-29 cells and this precedes activation of NF-*κ*B as ERK activation is seen from 24 h onwards (Elder *et al*, 2002). One target of ERK activity is the p90 ribosomal S6 kinase (p90^rsk^), a serine/threonine kinase that can phosphorylate I*κ*B*α* at serine 32 (Ghoda *et al*, 1997; Schouten *et al*, 1997). Therefore, activation of NF-*κ*B by NS-398 may involve the MEK/ERK pathway.

Several possible explanations may account for the lack of NF-*κ*B-dependent transcription in response to NS-398. For example, NS-398 may induce a protein or proteins that can inhibit the transcriptional activity of NF-*κ*B without interfering with DNA binding. Activation of NF-*κ*B DNA binding but not transcriptional activity, has been observed in response to both UV light and the anthracycline MEN 10755 (Campbell *et al*, 2001, Camarda *et al*, 2002). In both cases the lack of NF-*κ*B transcriptional activity could be attributed to increased levels of wild-type p53 protein leading to competition for the transcriptional coactivator proteins CBP/p300 (Webster and Perkins, 1999). This mechanism is, however, unlikely to be relevant here as HT-29 cells express high levels of mutant p53 (Rodrigues *et al*, 1990). Par-4 and c-myc have also been reported to inhibit NF-*κ*B at the level of transcription but not DNA-binding (Nalca *et al*, 1999; You *et al*, 2002). However, we were unable to demonstrate a significant increase in expression of either of these proteins following NS-398 treatment of HT-29 cells (results not shown), suggesting that the lack of transcriptional activity of NS-398-induced NF-*κ*B is not accounted for by an induction of these proteins. It has recently become apparent that NF-*κ*B activity is controlled not only at the level of I*κ*B degradation but also at the level of transcriptional activity, involving the direct post-translational modification of NF-*κ*B proteins. For example, protein kinase A (PKA) is reported to phosphorylate p65 at serine 276, enhancing the interaction of p65 with the transcriptional coactivator CBP/p300 (Zhong *et al*, 1998). Hence, it is possible that although NS-398 induces NF-*κ*B DNA-binding, it may be unable to stimulate the post-translational modifications of NF-*κ*B subunits required for full transcriptional activity.

In summary, our study demonstrates that, while capable of activating NF-*κ*B DNA binding, the NSAID NS-398 does not increase NF-*κ*B-dependent transcriptional activity in colon cancer cell lines. This suggests that, in contrast to apoptosis induced by other agents, activation of NF-*κ*B is unlikely to limit apoptosis induced by NS-398. The lack of induction of NF-*κ*B transcriptional activity by NS-398 is a promising outcome for the use of COX-2-selective NSAIDs not only in chemoprevention but also as novel therapies. The inability of NS-398 to increase NF-*κ*B-dependent transcription may be of particular importance when considering the use of COX-2-selective inhibitors in combination with other therapeutic agents, particularly those that act through the induction of apoptosis.
